# Legislative and judicial responses to workplace sexual harassment in mainland China: Progress and drawbacks

**DOI:** 10.3389/fpubh.2022.1000488

**Published:** 2022-09-26

**Authors:** Hao Wang

**Affiliations:** Shen Junru Law School, Hangzhou Normal University, Hangzhou, China

**Keywords:** workplace sexual harassment, mainland China, legislation, case rulings, gender equality

## Abstract

**Background:**

China has recently upgraded its anti-sexual harassment laws and regulations. The first-ever Chinese Civil Code, which took effect in 2021, has explicitly defined sexual harassment and imposed affirmative duties on employers to prevent and correct work-related sexual harassment. This study aims to map the status quo of China's anti-sexual harassment legal system and explore its progress and limits in dealing with workplace sexual harassment.

**Methods:**

We reviewed China's anti-sexual harassment laws at the national, provincial, and municipal levels and observed how they were enforced in courts. All judicial cases of workplace sexual harassment published by Chinese courts between January 2021 and June 2022 were examined. From a comparative law perspective, we then identified the progress and drawbacks of China's legislative and judicial responses to workplace sexual harassment.

**Results:**

China's current anti-sexual harassment legal system, while have made commendable progress, has its drawbacks: the definition of sexual harassment remains to be clarified and expanded to make it clear that sexual harassment is a form of gender discrimination and can include hostile environment harassment that is not directed against a specific person; the employer's obligations to prevent and correct sexual harassment need further delineation; employers lack guidelines for establishing a fair and effective grievance procedure; the difficulty of proving sexual harassment in litigation remains unsolved; the employer liability doctrine for sexual harassment lacks clarity; workers not in a traditional employment relationship receive inadequate legal protection from work-related sexual harassment.

**Conclusions:**

The issues mentioned above merit consideration in China's future law revisions and judicial practice. In China and other societies where gender inequality remains high, it is recommended to regulate sexual harassment as a form of discrimination and to set clear compliance standards for employers in preventing and correcting sexual harassment.

## Introduction

On August 7, 2021, an employee of Alibaba, one of China's e-commerce giants, posted online about being molested by a client and raped by her manager after a “drunken night”. She initially complained to Alibaba's human resources department, only to be disappointed that the company did not take action (1). After their probe, the police dropped the investigation into the alleged rape for lack of evidence. Still, they did arrest the client and the manager for “forcible indecency”,- which means molesting a victim through violence, coercion, or other means under Chinese law (2). As part of the Chinese #Metoo movement, the Alibaba scandal reignited national attention to work-related sexual harassment. *Peoples' Daily*, a state-backed media, also published an article to condemn toxic corporate cultures (3).

Sexual harassment is a public health (4) and human rights issue (5) in China and globally (6–9). Deeply rooted in gender inequality and sexism (10–12), sexual harassment affects women disproportionately in a patriarchal society (13–16). Sexual harassment often occurs in the workplace and can injure victims physically and mentally (17, 18), lower job satisfaction (19), increase job stress (20), harm human dignity, cause economic losses (21, 22), and prevent women from achieving their full potential in their careers (23). *Convention Concerning the Elimination of Violence and Harassment in the World of Work*, which entered into force on June 25, 2021, has recognized that everyone shall enjoy the right to a world of work free from gender-based harassment.

Combating sexual harassment requires multi-sectoral intervention, including enacting and enforcing anti-harassment laws. A social engineering tool, laws can regulate human behaviors and shape society (24). Currently, many countries have anti-sexual harassment laws that generally prohibit two types of workplace sexual harassment. One is *quid pro quo* harassment, that is, demanding sexual favors in return for some job benefits, which often occurs between supervisors and subordinates. The other is hostile environment harassment, which creates a hostile or intimidating environment for others (25–28). Hostile environment harassment does not have to be directed at a specific person—openly telling sexual jokes or posting pornographic posters in the workplace can constitute sexual harassment.

Though laws against workplace sexual harassment worldwide vary from country to country, there are common issues to be explored. First, how to define sexual harassment properly? Should the law regulate sexual harassment as a form of gender discrimination, a violation of human dignity, or both? Second, what reasonable measures should employers be legally required to take to prevent and correct workplace sexual harassment? Third, how to alleviate the victim's difficulty in proving a sexual harassment case without unduly sacrificing the interests of the accused? Fourth, as technology development and the COVID-19 pandemic have shifted the dynamics of the labor market (29, 30), how to ensure a future of work free from harassment?

In mainland China, sexual harassment emerged as a research topic in the mid-1990s, as the 1995 Fourth United Nations World Conference on Women held in Beijing helped increase awareness of violence against women. In the same year of the conference, a survey proved that sexual harassment was a real issue in China (31). Since then, Chinese scholars have explored the prevalence, causes, and effects of sexual harassment (32–35), including workplace sexual harassment (36–38).

During the last two decades, Chinese legal scholars have discussed the legal definition of sexual harassment (39–42), the protected interests of anti-harassment rules (43–45), and the employer liabilities of workplace sexual harassment (46). Compared to theoretical endeavors, legislative and judicial responses relatively lagged. Not a single sexual harassment case had entered judicial proceedings in mainland China before 2001 (47). It was not until 2005 that the first national law prohibiting sexual harassment was passed. Moreover, earlier anti-harassment laws in China were underdeveloped and under-enforced (48–50). The percentage of Chinese workers who had been harassed remained high in 2018 (51). Data from the 2010s revealed that many victims of workplace sexual harassment in China were still afraid to speak up—although formal public opinions in China seemed to oppose sexual harassment, informal ones still regarded being sexually harassed as a shame (52). Even if the victims were brave enough to sue, due to the lack of evidence and inadequate legislation, it was very difficult for them to win (53).

Has anything changed in recent years? Arguably, the Chinese #Metoo movement, which started with a college student accusing a professor of sexual misconduct in 2018 (54) and burst into the spotlight when a young woman filed a claim against a household-name celebrity in 2020 (55), has furthered public awareness of sexual harassment in China. Faced with public outcry, the Chinese authorities have signaled a commitment to fight against sexual harassment in recent years. The *Outline for the Development of Chinese Women (2021–2030)* issued by the State Council has set goals and missions to eliminate gender discrimination in employment (56). China's *National Human Rights Action Plan (2016–2020)* emphasized the need to “prevent and stop sexual harassment against women” (57). Since 2019, China's Supreme People's Court has recognized “sexual harassment disputes” as an independent cause of action, expressing its emphasis on anti-sexual harassment law enforcement (58). The first-ever Chinese *Civil Code*, which took effect in 2021, has introduced a series of new rules to address workplace sexual harassment.

What measures has China taken to address the common issues of sexual harassment law around the world? Can China's current laws and regulations curb sexual harassment in the workplace? This article aims to map the status quo of the Chinese anti-harassment legal system and compare it with international standards.

## Methods

To trace the evolution of China's anti-sexual harassment legislation, we conducted a full-text search of the keyword “sexual harassment” in the laws and regulations section of the *Chinalawinfo* database. Given that local legislation may not strictly follow national policies due to China's complex central-local relations (59), we searched for both national and local statutes at the provincial and municipal levels. In this way, we retrieved three national and 65 local statutes regarding sexual harassment. We conducted a legal textual analysis of these documents to examine how they define sexual harassment and employers' obligations in preventing and correcting sexual harassment at work.

To gain an understanding of how anti-sexual harassment laws on the books work in practice, we searched the *China Judgments Onlin*e, the official judicial judgment database established in mainland China, for case rulings published between January 2021 (when the *Chinese Civil Code* and its new anti-sexual harassment provisions took effect) and June 2022 with the keyword “sexual harassment”. Through a manual review of the retrieved judgments, we found that a total of 43 civil legal decisions (arising from 34 disputes) involving workplace sexual harassment were published in mainland China during the given period. By observing these cases' facts, issues, and holdings, we analyzed how Chinese courts defined sexual harassment, set the standard of proof of a sexual harassment claim, and interpreted the employer's legal obligations and potential liabilities.

Then we assessed the progress and deficiencies in China's anti-sexual harassment laws and their judicial enforcement with a comparative law perspective. Finally, we proposed how to improve China's anti-sexual harassment legal system to promote a harassment-free workplace. We also explored how our suggestions can be generalized to other places.

## Developments of anti-sexual harassment legislation in mainland China

### Legislation at the national level

In China, the National People's Congress and its Standing Committee have the power to make national laws and may authorize the State Council to enact administrative regulations according to actual needs. Based on the Constitution, following national laws and administrative regulations were passed to address sexual harassment (as summarized in Table 1).

**Table 1:** Anti-sexual harassment laws and regulations at the national level in China.

**Title**	**Issuing authority**	**Enactment date**	**Amendment date**	**Provisions governing sexual harassment**	**Rules specifying employer liability for harassment**
Women's Rights Protection Law	National People's Congress	1992-04-03	2005-08-28; 2018-10-26	Article 40 Sexually harassing women is prohibited. Victimized women have the right to complain to the employer and relevant organs.	None
The Special Regulations on Labor Protection of Female Workers	State Council	2012-04-18	-	Article 11 In the workplace, the employer shall prevent and stop the sexual harassment of female employees.	None
The Civil Code	National People's Congress	2020-05-28	-	Article 1010 If sexual harassment is carried out by means of verbal remarks, written language, images, physical acts, etc. against the will of others, the victim has the right to request the perpetrator to bear civil liability in accordance with the law. The State organs, enterprises, schools, and other similar organizations shall take reasonable precautions, hear, investigate and handle complaints, and take other like measures to prevent and stop sexual harassment conducted by a person through taking advantage of his/her position or a superior-subordinate relationship, and the like.	None

#### Women's Rights Protection Law (2005)

*Women's Rights Protection Law* with its amendment in 2005, was the first national law in mainland China to explicitly declare sexual harassment unlawful. Article 40 of the *Women's Rights Protection Law* prohibits “sexual harassment of women” and affirms that “sexual harassment victims have the right to complain to employers or relevant departments.” Questions are, are employers legally obligated to prevent sexual harassment? What should an employer do after receiving the complaint? Does the victim have the right to receive compensation from the employer? The *Women's Rights Protection Law* leaves these questions open.

#### The Special Regulations on Labor Protection of Female Workers (2012)

Article 3 of *China's Labor Law* (first enacted in 1995) has long recognized that “all workers, regardless of their genders and races, shall enjoy the right to equal employment and the right to occupational safety and health protection”. Whether this article prohibits workplace sexual harassment depends on a judge's interpretation. In 2012, China's State Council passed the *Special Regulation on Labor Protection of Female Workers*, which was the first national regulation in China to explicitly impose a duty on employers to “prevent and stop sexual harassment against female workers” (Article 11). However, whether the employers may demote or dismiss the harassers remains open.

#### The Civil Code (2020)

The first *Civil Code* in China, which was enacted on May 28, 2020 and took effect on January 1, 2021, aims to clarify, integrate, and amend existing rules in the field of Chinese private law. Regarding sexual harassment as damaging human dignity, the *Civil Code* has brought about noteworthy changes.

To begin with, the *Civil Code* is China's first national law to define sexual harassment explicitly. Its article 1020(1) states that sexual harassment is “performed in the forms of verbal remarks, texts, images, physical conducts, etc., against the will of others”. This definition has not clarified whether the law prohibits hostile environment harassment that does not target a specific victim. It is worth noting that, unlike earlier laws, the *Civil Code* uses gender-neutral language, meaning China's sexual harassment law is no longer only about women's rights.

Furthermore, the *Civil Code* codifies the obligations of employers. Its article 1010(2) requires employers to “take precautions, hear complaints, investigate and handle the cases, and take other like reasonable measures to prevent and stop sexual harassment, committed by a person through taking advantage of his or her position or a superior-subordinate relationship, and the like”. Just as article 1010(1), article 1010(2) seems to neglect hostile environment harassment, only emphasizing the need to prevent *quid pro quo* harassment committed through “taking advantage of one's position”.

### Legislation at the local level

According to China's *Legislation Law*, local legislative organs may formulate local laws, provided such laws do not contradict the Constitution, the national laws, and the administrative regulations (Article 63). Governments of provincial regions and larger cities may formulate local regulations in accordance with national laws, administrative regulations, and local laws (Article 73).

Although local legislation has received less attention from scholars, it is essential to China's anti-harassment legal system. In fact, Hubei province was the first in mainland China to explicitly prohibit sexual harassment in 1994, 11 years earlier than the national *Women's Rights Protection Law*.

As of June 2022, among 31 provinces in mainland China, 24 provinces have passed at least one anti-harassment law or regulation. Through the *Chinalawinfo* database, one can find 65 (54 provincial and 11 municipal) effective local laws or regulations containing the term sexual harassment, 50 (42 provincial and eight municipal) of which provide specific rules against workplace sexual harassment (Table 2). Based on these legal documents, this article analyzes how local legislation in China defines sexual harassment and sets out the obligations and liabilities of employers.

**Table 2:** Local legislation addressing workplace sexual harassment in China.

	**Title**	**Enactment date**	**Authority level**	**Contain rules on employer obligation to address sexual harassment**	**Contain rules specifying employer liability for harassment**
1	Regulations on Labor Protection of Female Employees in Heilongjiang Province	2021-08-23	Provincial laws	Yes	No
2	Regulation on Labor Union Labor Law Enforcement Supervision in Shandong Province	2021-07-29	Provincial laws	No	No
3	Liaoning Provincial Rules on Implementation of the Women's Rights Protection Law of the People's Republic of China (2020 Amendment)	2020-11-25	Provincial laws	Yes	No
4	Regulations on the Protection of Women's Rights and Interests of Jiangsu Province (2020 Amendment)	2020-07-31	Provincial laws	Yes	No
5	Fujian Provincial Regulations on Labor Protection of Female Employees	2020-03-20	Provincial laws	Yes	No
6	Regulations on the Protection of Women's Rights and Interests of Ningxia Hui Autonomous Region (2019 Amendment)	2019-07-27	Provincial laws	Yes	No
7	Measures of Shanxi (陕西) Province for Implementing the Law of the People's Republic of China on the Protection of Women's Rights and Interests (2019 Amendment)	2019-07-31	Provincial laws	Yes	No
8	Regulations of Hebei Province on the Protection of Women's Rights and Interests	2017-07-28	Provincial laws	Yes	No
9	Tianjin Regulations on the Protection of Women's Rights and Interests	2016-11-18	Provincial laws	Yes	No
10	Measures for Guangxi Zhuang Autonomous Region to Implement the Women's Rights Protection Law of the People's Republic of China (2010 Amendment)	2010-07-30	Provincial laws	Yes	No
11	Measures of Beijing Municipality to Implement the Women's Rights Protection Law of the People's Republic of China (2009 Amendment)	2009-09-25	Provincial laws	Yes	No
12	Measures of Qinghai Province to Implement the Women's Rights Protection Law of the People's Republic of China (2009 Amendment)	2009-09-24	Provincial laws	Yes	No
13	Measures for Shandong Province to Implement the Women's Rights Protection Law of the People's Republic of China (2009 Amendment)	2009-01-08	Provincial laws	Yes	No
14	Several Provisions on the Protection of Women's Rights and Interests in Hainan Province	2008-12-01	Provincial laws	Yes	No
15	Measure for Inner Mongolia Autonomous Region to Implement the Women's Rights Protection Law of the People's Republic of China	2008-11-04	Provincial laws	Yes	No
16	Measures for Henan Province to Implement the Women's Rights Protection Law of the People's Republic of China	2008-09-26	Provincial laws	Yes	No
17	Measures for Yunnan Province to Implement the Women's Rights Protection Law of the People's Republic of China (2008 Amendment)	2008-9-25	Provincial laws	Yes	No
18	Measures for Shanxi (山西) Province to Implement the	2007-12-10	Provincial laws	Yes	No
19	Measures for Jilin Province to Implement the Women's Rights Protection Law of the People's Republic of China (2007 Amendment)	2007-11-30	Provincial laws	Yes	No
20	Measures of Gansu Province to Implement the Women's Rights Protection Law of the People's Republic of China (2007 Amendment)	2007-09-27	Provincial laws	Yes	No
21	Measures of Sichuan Province to Implement the Women's Rights Protection Law of the People's Republic of China (2007 Amendment)	2007-09-27	Provincial laws	Yes	Yes
22	Measures of Hubei Province to Implement the Women's Rights Protection Law of the People's Republic of China (2007 Amendment)	2007-07-28	Provincial laws	Yes	No
23	Measures of Zhejiang Province to Implement the Women's Rights Protection Law of the People's Republic of China (2007 Amendment)	2007-07-26	Provincial laws	Yes	No
24	Measures of Guangdong Province to Implement the Women's Rights Protection Law of the People's Republic of China (2007 Amendment)	2007-05-31	Provincial laws	Yes	No
25	Measures of Shanghai Municipality to Implement the Women's Rights Protection Law of the People's Republic of China (2007 Amendment)	2007-04-26	Provincial laws	Yes	No
26	Measures of Guizhou Province to Implement the Women's Rights Protection Law of the People's Republic of China (2007 Amendment)	2007-03-30	Provincial laws	Yes	No
27	Measures of Heilongjiang Province to Implement the Women's Rights Protection Law of the People's Republic of China	2006-10-20	Provincial laws	Yes	No
28	Measures of Hunan Province to Implement the Women's Rights Protection Law of the People's Republic of China	2006-07-31	Provincial laws	Yes	No
29	Measures of Xinjiang Uygur Autonomous Region to Implement the Women's Rights Protection Law of the People's Republic of China	2006-05-25	Provincial laws	Yes	No
30	Special Regulations on Labor Protection for Female Workers in Guizhou Province	2021-12-31	Provincial regulations	Yes	No
31	Labor Protection Measures for Female Employees of Liaoning Province	2020-12-09	Provincial regulations	Yes	No
32	Special Regulations on Labor Protection for Female Workers in Hunan Province	2019-12-09	Provincial regulations	Yes	No
33	Special Regulations on Labor Protection for Female Workers in Jiangxi Province (2019 Amendment)	2019-09-29	Provincial regulations	Yes	No
34	Special Regulations on Labor Protection for Female Workers in Shandong Province	2019-01-16	Provincial regulations	Yes	No
35	Special Regulations on Labor Protection for Female Workers in Henan Province	2018-09-06	Provincial regulations	Yes	No
36	Special Regulations on Labor Protection for Female Workers in Jiangsu Province	2018-05-08	Provincial regulations	Yes	No
37	Special Regulations on Labor Protection for Female Workers in Shanxi (陕西) Province	2018-01-12	Provincial regulations	Yes	No
38	Special Regulations on Labor Protection for Female Workers in Jiangxi Province	2017-05-19	Provincial regulations	Yes	No
39	Special Regulations on Labor Protection for Female Workers in Zhejiang Province	2017-04-14	Provincial regulations	Yes	No
40	Measures for Guangdong Province to Implement the Special Regulations on Labor Protection of Female Workers	2016-12-20	Provincial regulations	Yes	No
41	Special Regulations on Labor Protection for Female Workers in Hebei Province	2016-11-18	Provincial regulations	Yes	No
42	Special Regulations on Labor Protection for Female Workers in Anhui Province	2016-01-27	Provincial regulations	Yes	No
43	Shenzhen Special Economic Zone Gender Equality Promotion Regulations (2019 Amendment)	2019-06-19	Municipal laws	Yes	No
44	Provisions on the Protection of Women's Rights and Interests in Guangzhou (2020 Amendment)	2020-08-20	Municipal laws	Yes	No
45	Provisions on the Protection of Women's Rights and Interests in Suzhou (苏州)	2020-08-10	Municipal laws	Yes	No
46	Provisions on the Protection of Women's Rights and Interests in Harbin	2014-09-10	Municipal laws	Yes	No
47	Provisions on the Protection of Women's Rights and Interests in Baotou	2012-05-10	Municipal laws	Yes	Yes
48	Provisions on the Protection of Women's Rights and Interests in Chengdu	2011-11-04	Municipal laws	Yes	No
49	Provisions on the Protection of Women's Rights and Interests in Jinan	2011-01-04	Municipal laws	Yes	No
50	Measures for Qingdao to implement to Implement the Women's Rights Protection Law of the People's Republic of China	2010-09-29	Municipal laws	Yes	No

#### The local definition of sexual harassment

Among local laws and regulations, two (Heilongjiang province in 2022 and Liaoning province in 2021) have adopted an explanation consistent with the *Civil Code*, saying sexual harassment is “performed in the forms of verbal remarks, texts, images, physical conducts, etc., against the will of others”.

Local legislation that predates the *Civil Code* provides various definitions. A considerable number of them state that sexual harassment must be “related to sex and involve sexual content” (Jiangsu province, 2020; Beijing municipality, 2009; Chongqing municipality, 2008; Anhui province, 2007; Guangdong province, 2008; Baotou municipality, 2020). Some adopt a narrower definition, saying that sexual harassment must be carried out in a way that “contains obscene content” (Qinghai province, 2009; Qinghai province, 2008; Hubei province, 2007; Shanxi province, 2007; Hunan province, 2006) or “sexual demands” (Shanxi province, 2019). If such narrow definitions are adopted, behaviors such as pressuring for dates will not constitute sexual harassment, as they contain no “obscene content” or direct “sexual demands”.

There is also local legislation that appears to be ahead of national laws. *Jiangsu Provincial Special Provisions on Labor Protection for Female Employees* (2018) and *Measures for the Implementation of the Law of the People's Republic of China on the Protection of Rights and Interests of Women in Guangdong Province* (amended in 2007) require employers to “ensure a working environment free from sexual harassment”, which is a step toward recognizing hostile environment harassment.

#### The employers' obligations imposed by local legislation

**Figure 1:**
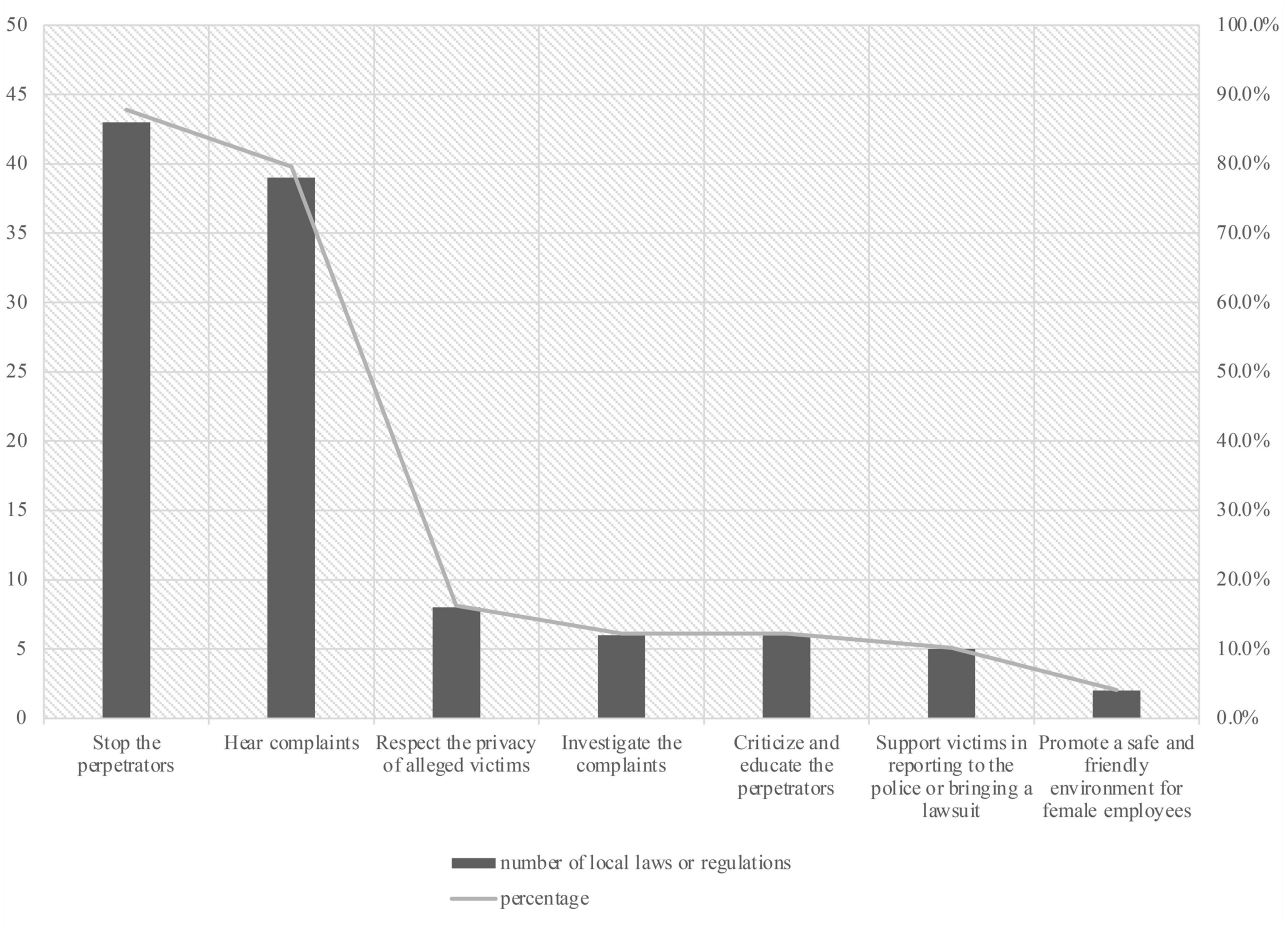
Employer obligations explicitly required by local laws and regulations in mainland China. Data current to July 17, 2022.

As Table 2 shows, of the 50 local statutes that mention workplace sexual harassment, 49 stipulate the obligations of employers. As Figure 1 indicates, among these 49 local statutes, two (4.1%) require employers to create a friendly environment for female employees. Thirty-nine (79.6%) stipulate that the employer should hear complaints from victims. Six (12.2%) require employers to initiate the investigation process after receiving complaints. Eight (16.3%) emphasize that employers shall respect the privacy of victims during the grievance procedure.

In the case of proven harassment behaviors, all 49 local laws and regulations involving employers' obligations require corrective measures. Forty-three (87.8%) mandate the employers to “stop the perpetrator”. Six (12.2%) state that employers shall “criticize and educate” the perpetrators. Notably, five provinces (Heilongjiang, Guizhou, Liaoning, and Shandong) and one municipality (Beijing) require employers to support victims in reporting to the police or bringing a lawsuit.

Local laws and regulations have further clarified employers' duties compared to national legislation. Still, many questions remain in the air. First, although many local laws and regulations require employers to handle complaints, most are expressed in vague terms such as “handle in accordance with law” or “handle in time”, without providing any practical guidelines for the grievance procedure. Second, local legislations have not stipulated whether the employer may demote or dismiss the perpetrators. Moreover, like national legislation, the vast majority of local legislations have not provided explicit provisions on employer liability. Only two local regulations *(Measures for the Implementation of the Women's Rights Protection Law of Sichuan Province*, revised in 2007; *Provisions on the Protection of Women's Rights and Interests in Baotou, 2012*) affirm that employers shall bear civil liability for workplace sexual harassment.

## Judicial enforcement of anti-harassment laws in Chinese courts: Evidence from case rulings

**Figure 2:**
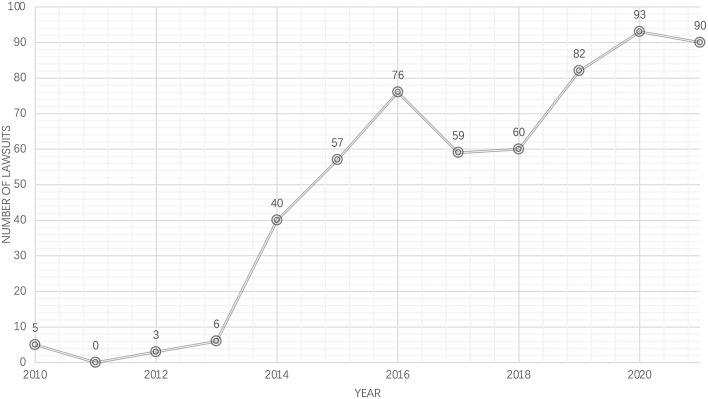
Number of lawsuits involving sexual harassment in Mainland China (2010–2021). Source: China Judgments Online; data current to July 17, 2022.

Laws on books and in practice can differ. Therefore, it is necessary to examine the relevant judicial rulings. Searching for “sexual harassment” on *China Judgments Online*, one can find that the number of civil lawsuits involving sexual harassment has shown a growing trend (Figure 2). As mentioned earlier, the *Civil Code*, which came into effect on January 1st, 2021, has introduced new provisions on sexual harassment in the workplace. To evaluate the impact of the recent reforms, we analyze the judicial rulings published in mainland China from January 2021 to June 2022. During the time, *China Judgments Online* published 93 judgments mentioning sexual harassment. Through a manual review, we retrieved a total of 34 judicial disputes and 43 corresponding judicial decisions involving workplace sexual harassment (some cases have undergone appeals). Based on the sample cases, we obtain the following observations.

### Who are litigating, and on what causes?

Although the anti-harassment provision in the *Civil Code* adopts gender-neutral language, all the alleged perpetrators in the sample cases are male, and all the alleged victims are female.

**Figure 3:**
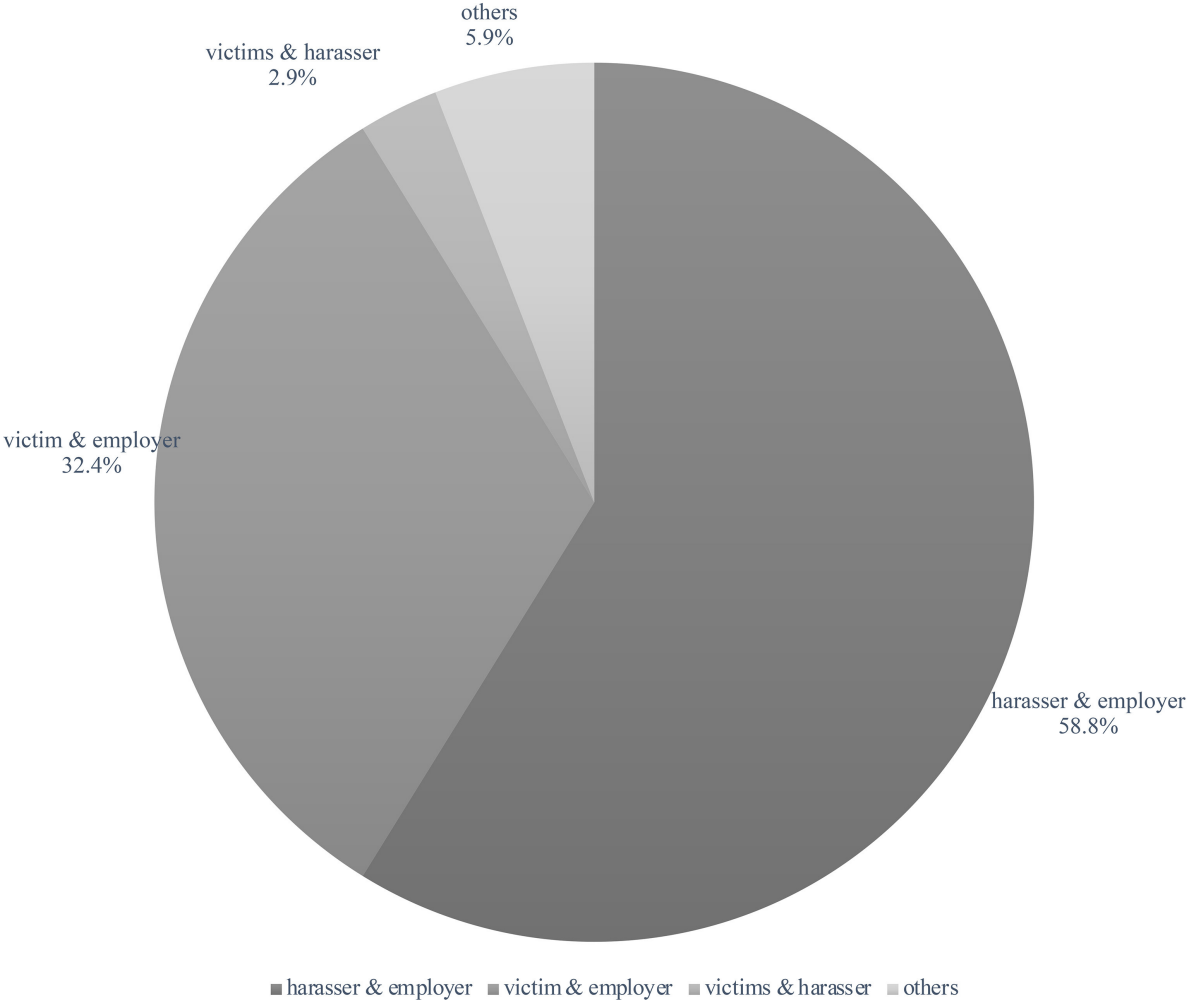
Parties of the lawsuits involving workplace sexual harassment (January 2021–June 2022). Source: case rulings published on China Judgments Online between January 2021 and June 2022; data current to July 17, 2022.

As shown in Figure 3, among the 34 sample disputes, 20 (58.8%) were between the alleged harasser and the employer, all of which happened because the employer had dismissed or otherwise sanctioned the alleged harassers. Two cases involved the whistleblowers (one whistleblower sued the employer for wrongful termination, and the other whistleblower was sued by the alleged harasser for defamation).

**Figure 4:**
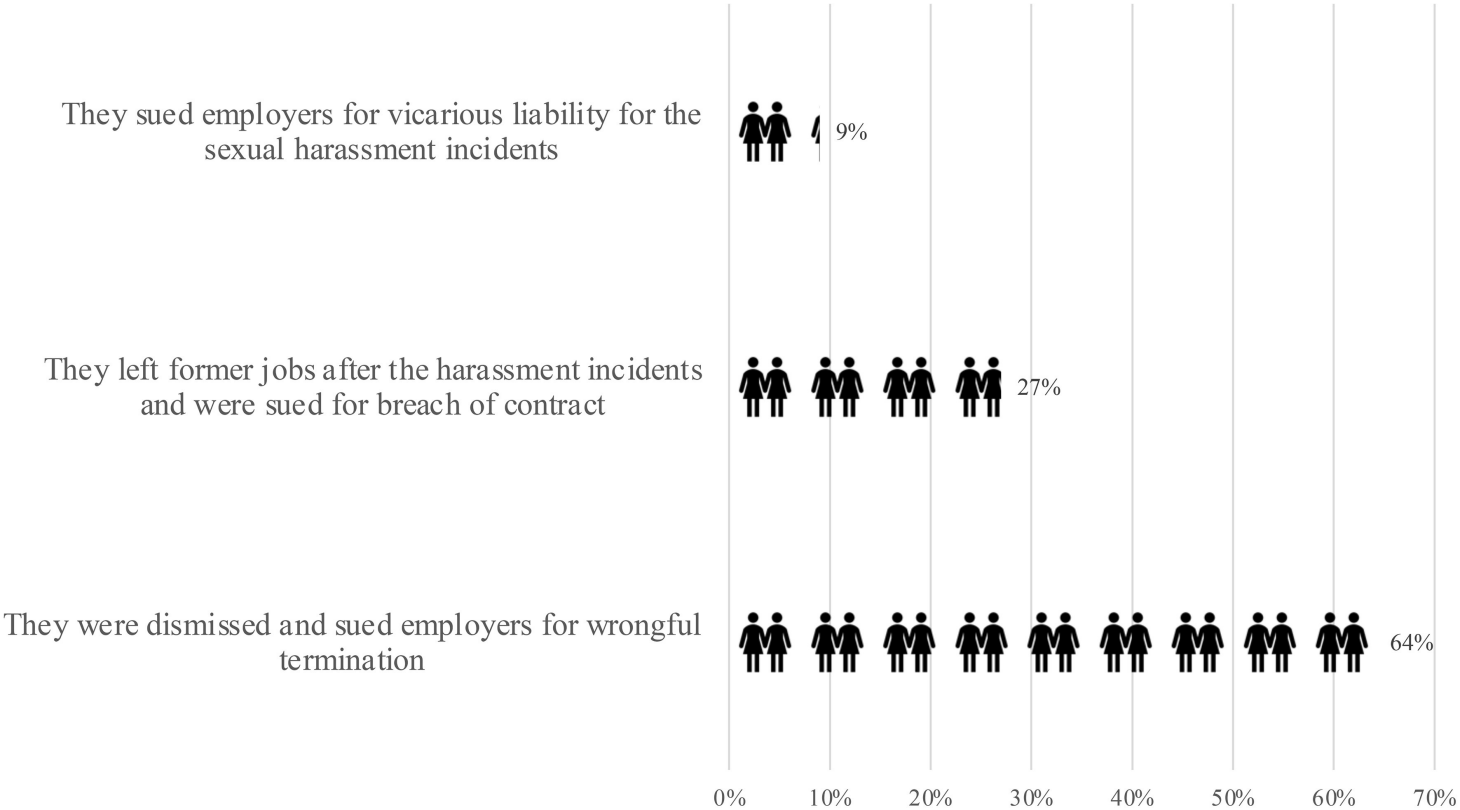
Why did the alleged victims participate in the lawsuits? Source: case rulings published on China Judgments Online between January 2021 and June 2022; data current to July 17, 2022.

Alleged victims participated in only 12 (32%) cases (11 against the employer and one against the alleged harasser). From the facts of these cases, victims of workplace sexual harassment are unlikely to sue unless they have left their former jobs. As Figure 4 shows, among the 11 sample disputes between the alleged victims and the employers, only one victim sued her employer for vicarious liability for sexual harassment. Seven alleged victims filed lawsuits, not for sexual harassment but for unfair dismissal. These alleged victims got fired and only brought up the sexual harassment claim in front of the judges during the wrongful termination lawsuits. In the other three cases, the employers sued the employee who resigned for breach of contract, while the employees contended that they had to leave because they had suffered workplace sexual harassment.

Not limited to victims of workplace sexual harassment, victims of sexual harassment in mainland China, in general, file fewer lawsuits. From January 2021 to June 2022, only six alleged victims brought sexual harassment claims against the alleged perpetrators, four of whom eventually withdrew their lawsuits. The *2018 China Workplace Sexual Harassment Survey Report* also shows that most (70.3%) sexual harassment victims choose not to speak up (51).

There are many reasons behind the victim's silence. First, the potential financial stress of leaving a job can deter employees from speaking up. As mentioned earlier, Chinese employees who choose to stay at the company tend not to file a workplace sexual harassment lawsuit. Second, traditionally, sex and sexuality were taboo in China, and “losing virginity” was considered degrading for unmarried women. Although Chinese sex culture has changed dramatically with the country's reform and opening up, the influence of traditional ideologies persists (60). Therefore, the experience of being sexually harassed could be considered shameful and negatively affect a female victim's subsequent relationships and marriages. Third, in a gender-unequal society, many Chinese women still lack awareness of their rights, leading to their higher tolerance of sexual harassment (61). In addition, according to the *2018 China Workplace Sexual Harassment Survey Report*, the fear of ruining work relationships and the fear of retaliation are two of the main reasons why victims choose not to report workplace sexual harassment, accounting for 46 and 30% of the respondents, respectively (51). The sample case studies in this article showed that retaliation against victims or whistleblowers does remain an issue. In *Tsubaki Motor Automotive Shanghai Co., Ltd. v. Zhou Xian* (62), an alleged victim filed a lawsuit against the company as she was fired for “calling the police with insufficient evidence.” *In Hou Bing v. China Minsheng Bank Co., Ltd*. (63), a whistleblower suffered retaliation from his employer. In both lawsuits, the court ruled against the employers. In the former case, the court emphasized the need to protect the employees' right to complain, stating that “one may report sexual harassment to the police even if there is insufficient evidence”.

### How do courts define sexual harassment?

In civil proceedings in mainland China, judges serve as fact-finders. Of the 43 court rulings arising from the 34 sample disputes, only two judgments specifically discussed the definition of sexual harassment. In *Zheng Shiqiang v. Zhuohua Clothing Ltd*. (64), the defendant dismissed the plaintiff for sexual harassment, including intentionally blocking female colleagues' way and making unwanted physical contact. The plaintiff argued that, as his behaviors did not involve a sexual act, he did not commit sexual harassment. As noted earlier, some local legislations in China do narrowly define sexual harassment, saying that it must include “obscene contents”. The court, however, ruled against the plaintiff and said that sexual harassment was “related to sex”, which “should not be construed so narrowly as involving a sexual act. In *Du v. Lu* (65), a hotel employee sued the head chef. While the employee failed to provide evidence of physical harassment, witnesses testified that the head chef “sometimes made sexual jokes around the workplace”. Although the *Civil Code* does not expressly prohibit hostile environment harassment not directed at a specific person, the court ruled in favor of the victim. It held that “the head chief, with his management power in the hotel, should have fully respected the dignity and inner feelings of female employees…to create a civilized and healthy working environment”.

It is worth mentioning that, unlike views of many jurisdictions around the world, none of the sample judgments has linked sexual harassment with sex discrimination.

### How to prove sexual harassment in courts?

In civil proceedings, Chinese courts adopt the principle of “whoever claims, who gives evidence”. The burden of proof is met when the party proves that a claim is highly probable to be true. This high probability standard is higher than the preponderance standard (“more likely than not” standard), which prevails in common law systems (66). As sexual harassment often happens in private and usually does not inflict apparent physical damage, it is notoriously difficult for victims to meet the high probability standard. In the sample cases, the evidence submitted to prove sexual harassment mainly included self-confession documents, surveillance videos, chat logs, police records, and witness testimonies. As described below, the courts did not agree on the adequacy of these different kinds of evidence.

- **Letters of apology and other similar documents of a self-confessed nature**

In six sample cases, alleged perpetrators had once promised not to commit sexual harassment again in written form as requested by the employer or the police after their investigation. In all these cases, the courts believed that the sexual harassment claim was substantiated (63–65, 67–70).

- **Surveillance videos**

Putting aside the possible privacy issues, a video capturing the harassment behavior could be critical evidence. However, the court might find the evidence insufficient if the recorded behavior was not of blatant sexual nature. In *Guangzhou Guanli Real Estate Agency Co., Ltd. v. Gao* (71), the court held that a video of the defendant touching a colleague's face was insufficient to prove the sexual harassment claim, given that the video failed to show apparent resistance from the colleague.

- **Chat logs**

The original copies of chat logs that contained harassing language or mentioned the harassment incidents could be used to prove harassment (68). Screenshots were deemed inadmissible (72–74).

- **Police records**

Some victims had reported to the police before litigations. If the police investigation supported the harassment claim, the police records were solid evidence as a matter of course. However, what if the police found insufficient evidence to pursue the harassment case? In *Jiang Zhengming v. Aidu Hotel* (75, 76), the court simply deferred to the conclusion of the police investigation. By contrast, in *Zheng Shiqiang v. Zhuohua Clothing Ltd*., the court held that the findings of the police did not necessarily affect the civil proceedings (64).

- **Witness testimonies**

Witnesses had a vital role to play. In *Du v. Lu* (65), two colleagues testified that the alleged perpetrator did like to make sexual jokes in the workplace. The court held in favor of the alleged victim, despite no tangible evidence.

In summary, surveillance videos, chat logs, and witness testimonies can be helpful, but obtaining such evidence is not always easy. As to police records, there is ambiguity regarding the appropriate level of deference the court should give to the police. Apology letters or other self-confession documents have high probative value. It is worth noting that most of these documents were written as requested by the employer or the police after their investigation. Therefore, support from the employer and the police is critical for victims.

### How do courts define employers' rights and duties?

As mentioned earlier, current Chinese laws mandate employers to take reasonable measures to prevent sexual harassment. However, it needs clarification on what measures are required and when the employers will be held vicariously liable for sexual harassment. Unfortunately, the sample cases barely discussed these issues, since the victim rarely brought vicarious liability claims against the employers.

Another related question is, does the employer have the right to take disciplinary actions, such as demotion and dismissal, against the perpetrators? From the 18 sample disputes involving employers and the alleged harassers, the courts' position is clear-as long as the court found the sexual harassment claim was substantiated, the legality of the employer to terminate the labor contract was recognized. It is notable that, in *Changchun Hengye Co., Ltd. v. Zhang Huanshan* (77), the court held that, even though the company's written policy did not contain an anti-sexual harassment clause, the company had the right to terminate the harasser. In *Huxiao v. Baoan Maternal and Child Health Hospital* (78), the court held that the hospital had the right to suspend an anesthesiologist after receiving a joint complaint from 36 female employees.

### A new challenge in digital economy: How do courts respond to workplace sexual harassment against social media influencers?

Two sample cases were exceptional in the sense that they involved “social network influencers”, who were not in a traditional employment relationship. In *Bian Culture Media Co., Ltd. v. Guo Yanfei* (79) and *Jiujia Culture Media Co., Ltd. v. Tian Wenqi* (80), the defendants were influencers who worked under the plaintiffs' management. They had stopped working after allegedly being sexually harassed by their managers and ended up being sued for breach of contract. The courts in both cases affirmed that the “collaboration contractual relationship” between the parties had been terminated. However, such court judgments were not entirely good news for the influencer community. As the courts denied the employer-employee relationship between the influencers and their management company, the company arguably had no obligation to prevent sexual harassment against influencers, and the influencers could lose protection from labor laws.

## Discussion

Many factors have contributed to the difficulties faced by victims of workplace sexual harassment in mainland China, including the underdeveloped anti-harassment laws, the inaction of the employers, the difficulty in gathering evidence, and the tremendous pressure from cultural ideologies regarding gender and sexuality (37, 81). Can the recent legal reforms help to alleviate these problems? This article suggests that, while recent reforms have made commendable progress, they still have many drawbacks.

### Progress

#### Legal definition of sexual harassment has been gradually formed and expanded

Before *Civil Code* (2021), the vague legal definition of sexual harassment allowed some Chinese judges to adopt narrow interpretations unfavorable to victims. For instance, in a case published in 2006 (82), a court held that a party repeatedly sending unwelcome texts, saying “it does not prevent me from thinking of you” and “let my yearns come true”, did not constitute sexual harassment because the messages were “not sexual enough”. Similarly, a case ruling published in 2016 said that a supervisor pinching an intern's nose and hugging her was not sexual harassment (83). In a lawsuit in 2017, the court denied the defendant had committed sexual harassment by repeatedly sending unwelcome text messages and secretly filming the plaintiff when she was dancing with others, since “the defendant did not use obscene language, nor did he intentionally touch any sexually sensitive parts” (84).

In recent rulings involving workplace sexual harassment, courts no longer define sexual harassment as narrowly as the above-cited judgments. The judgment of *Zheng Shiqiang v. Zhuohua Clothing Ltd*. pointed out that sexual harassment did not necessarily involve explicitly sexual behavior (64). Despite the absence of national legislation that explicitly prohibits hostile environment harassment, a court ruled that making sexual jokes in the workplace, even if not targeted at a specific person, counted as sexual harassment (67).

Another notable advance is the adoption of gender-neutral language in the anti-sexual harassment provisions of the *Chinese Civil Code*, confirming that anti-sexual harassment laws protect everyone, regardless of gender.

#### The laws and regulations have recognized the employers' obligations to take preventive and corrective measures

Employers' intervention is widely acknowledged to be essential in combating workplace sexual harassment (85). Unfortunately, according to a 2009 survey conducted in mainland China, victims were often ignored or even retaliated against by their employers (37). For example, after making a complaint, one of the survey respondents received a reply saying, “text flirting and touching butt…are they even sexual harassment?” Another respondent was told by the employer to “check your own behavior first (37)”.

As mentioned earlier, current laws and regulations in mainland China, both nationally and locally, have recognized the obligation of employers to prevent and correct sexual harassment. Some local statutes explicitly require employers to support the victims' lawsuits, which may help to mitigate the difficulties of proving sexual harassment in subsequent litigation. In terms of corrective measures, the courts in mainland China have generally recognized that the employer has the right to take disciplinary actions, including demotion and dismissal, which will help to deter sexual harassment in the workplace.

#### Some courts have responded to the difficulties in proving sexual harassment

By examining recent court rulings, this article notes that some judges in mainland China have been aware of the problem that sexual harassment cases can be very difficult to prove. In *Tsubaki Motor Automotive Shanghai Co., Ltd. v. Zhou Xian*, when an employer dismissed an alleged victim for “calling the police without sound evidence”, the court emphasized that an employee who believed she had been harassed had the right to make a report to the police even without sufficient evidence (62). In *Huxiao v. Baoan Maternal and Child Health Hospital*, the courts held that the claimed sexual harassment was highly probable based on a joint letter issued by 36 female colleagues of the alleged perpetrator, even if there was no tangible evidence (86). These rulings can help to provide a friendly court environment for work-related sexual harassment survivors in mainland China.

### Drawbacks

#### The legal definition of sexual harassment still lacks clarity and needs expansion

In the history of the development of anti-sexual harassment laws and regulations, different countries have different understandings of sexual harassment at different times. One approach interprets sexual harassment as a form of gender discrimination, another considers sexual harassment as a violation of personal rights (right to personal dignity or physical integrity), and a third is a mixture of the “discrimination approach” and the “dignity approach” (87, 88).

Mainland China has adopted the “dignity approach”. As analyzed above, the *Chinese Civil Code* regards sexual harassment as dignitary harm, and the Chinese court has not defined sexual harassment as gender discrimination. Similarly, Malaysia's *Anti-Sexual Harassment Bill 2021* also adopts the dignity model by defining sexual harassment as “any unwanted conduct of a sexual nature, in any form, whether verbal, non-verbal, visual, gestural or physical, directed at a person which is reasonably offensive or humiliating or is a threat to his wellbeing” (89).

Different from China, the United States takes the “discrimination approach”. US courts have conceptualized workplace sexual harassment as a form of employment discrimination, actionable based on the Title VII of the *Civil Rights Act of 1964*. Under this approach, any harassment “because of sex” constitutes sexual harassment, even if not of sexual nature (90).

In some regions, there has been a shift in how sexual harassment is conceptualized. The EU Law traditionally adopted a “dignity approach”, but it has turned to a mixed approach, regulating sexual harassment as both dignitary harm and gender discrimination (91). Accordingly, some European countries, such as France, have gradually changed how they define sexual harassment (87). In Asia, Japan has regulated sexual harassment as gender discrimination since 2006 (92). In its newly enacted *Protection against Harassment of Women at the Workplace (Amendment) Act 2022*, Pakistan also extended the definition of harassment to gender-based harassment for the first time (93).

China's pure “dignitary approach” has its drawbacks. First, focusing more on individual harm than on systemic inequality, the “dignity approach” tends to narrow the definition of sexual harassment by ignoring that sexual harassment stems from structural power disparities and may not always have a sexual nature. As shown by the local statutes, sexual harassment behaviors are often considered solely a sexual problem in China, which must have a sexual nature or even “obscene contents”. However, in the workplace in China, harassment related to sex is not necessarily aimed at sexual cooperation. A survey revealed that 21.1% of Chinese LGBTI respondents had experienced workplace harassment, bullying, or discrimination due to their sexual orientation; ~41% of them found their workplace “not open and intolerant” (94). With its “discrimination approach”, the U.S. case law has long declared that actionable sexual harassment need not be motivated by sexual desire (95). Therefore, in *Franchina v. City of Providence*, the court affirmed that a heterosexual man harassing a homosexual woman based on sexual orientation is sexual harassment (96). These rulings echoed Vicki Schultz's argument more than 20 years ago: sexual harassment was about sexism and did not necessarily stem from sexual desire (12). If the same case had happened in China, the court would probably not have made the same judgment.

Secondly, in a society where gender inequality remains a serious issue, the “dignity approach” may further solidify gender stereotypes- where sexism in a variety of forms is tolerated, behaviors that objectify women or discriminate against sexual minorities are often seen as normal and innocuous, instead of dignity-harming. For example, a high-profile lingerie advertisement in the Chinese market still blatantly suggests that female workers would often use sex to get a promotion by saying, “this is a ‘career lifejacket’ that lets you win the workplace by simply ‘lying down”’ (97).

Why has the *Chinese Civil Code* not explicitly prohibited creating hostile, intimidating, or offensive working environments for others, while many other jurisdictions worldwide [for example, the United States (25), the EU (26), Pakistan (27), India (28), and the Hong Kong SAR of China (98)] have done so? One of the main reasons may be that, in Chinese workplace culture, many behaviors that may constitute hostile sexual harassment are often considered acceptable and even plausible ways to maintain a lively work atmosphere. According to the *2018 China Workplace Sexual Harassment Survey Report*, “asking about or talking about sexual experiences against the will of others” (61.3%) and “making offensive sexual jokes in the office or at dinner parties” (48.7%) were the most common forms of workplace sexual harassment (51). However, the report showed that only 57.1% of male respondents thought that telling sexual jokes may constitute sexual harassment (51); 28.6% of female and 26.9% of male respondents did not consider repeatedly pressuring for dates as a form of sexual harassment (51). In addition, while inappropriate staring and leering could lead to an intimidating environment (99), a psychological expert said during an interview: “The definition of sexual harassment should be strict...Staring at others means one has poor cultural, moral, and psychological qualities. However, it could not be included in the legal scope of sexual harassment (100).” In mainland China, it is also not uncommon for enterprises to use sexually suggestive games as team activities “to promote team friendship.” Some female employers of Alibaba, the company mentioned at the beginning of the article, once revealed in an interview that they felt “embarrassed and degraded” by being forced to participate in games with sexual connotations (101). In a workplace that tolerates sexual harassment, women who feel uncomfortable may be considered overly sensitive, and some women may not even realize that their rights have been compromised and resist the implementation of sexual harassment regulations as men do (102).

The *General Recommendation 19 of Elimination of All Forms of Discrimination Against Women* issued by the UN committee has long recognized sexual harassment as a form of discrimination. Viewing sexual harassment as gender discrimination is recommended since the role of anti-discrimination laws is to challenge the current social norm by promoting a new consensus on acceptable behaviors. Therefore, Chinese policymakers shall clarify and expand the legal definition of sexual harassment to at least explicitly prohibit hostile environment harassment and preferably reframe sexual harassment as both dignitary harm and discrimination wrongs.

#### The employers lack guidance regarding their duties to prevent and correct sexual harassment

Organization tolerance can increase the occurrence of work-related sexual harassment (103). Thus, it is significant progress that the current national laws in China have required employers to take reasonable measures to prevent and correct sexual harassment in the workplace. However, many questions are left open. For example, must employers formulate a written anti-sexual harassment policy? Is employee training on sexual harassment mandatory?

Regarding employers' obligations to prevent workplace sexual harassment, U.S. case law mainly focuses on whether the organization has formal anti-harassment policies, provides sexual harassment training to employees, and has a proper grievance procedure (104). This approach encourages employers to comply with a series of pre-set standards. In some legal systems, statutory laws explicitly prescribe compliance requirements. For example, section 19 of India's *The Sexual Harassment of Women at Workplace (Prevention, Prohibition and Redressal) Act* (2013) requires employers to “display at any conspicuous place in the workplace, the penal consequences of sexual harassment” and “organize workshops and awareness programs at regular intervals for sensitizing the employees” (28).

Although some scholars regard the formulation of written policies, regular training, and the establishment of internal complaint mechanisms as measures enterprises should take (105), some legal scholars criticize these measures as symbolic. These scholars believe that by focusing on whether the employers' actions comply with some standardized requirements, instead of assessing the effectiveness of the efforts, the “compliance approach” is an unfortunate triumph of “form over substance”, discouraging employers from doing more than the minimum compliance standards (106).

Despite its limitations, establishing clear compliance standards is recommended for mainland China, where there is still an urgent need to raise employers' awareness about sexual harassment at work. Although China has required employers to prevent and stop sexual harassment since 2012, employers are doing far from enough to combat sexual harassment. According to a survey released in 2018, few Chinese employers have formulated anti-harassment policies; many employers fail to investigate sexual harassment complaints, let alone take corrective measures (51). In the case mentioned at the beginning of this article, Alibaba, one of China's largest companies, also failed to handle sexual harassment complaints promptly. At this stage, legislation or judicial judgments shall provide employers with more guidance on what measures are mandatory and what are best practices by setting clear standards of compliance, without creating undue legal uncertainty and overburdening employers.

#### The employers lack guidance on ensuring procedural fairness when handling complaints

While providing speedy remedies for victims is essential, it must be noted that not all allegations are true. Employers shall establish a fair, impartial, and transparent procedure for both the claimant and the accused. According to the *Guidebook for the Prevention and Control of Sexual Harassment in the Workplace* issued by the All-China Women's Federation in 2021, employers are recommended to take the following steps in a grievance procedure: hearing complaints; investigating the cases; making decisions; reviewing objections; notifying results (107).

Unfortunately, seen from the sample cases, few employers had adopted a formal procedure for handling sexual harassment complaints. While recognizing the employer's right to sanction the perpetrator, judges rarely looked into the accused's due process right in court rulings. So far, employers in mainland China still lack official guidance for complaint handling procedures.

#### The rules of employer liability for workplace sexual harassment remain to be established

Potential liabilities should back obligations. For example, according to U.S. caw law, the employers will be held strictly liable, if the harassment results in a tangible employment action, such as termination of employment or promotion. If there is no tangible employment action, the employer shall only bear vicarious liability when it fails to prove: (1) it exercised reasonable care to prevent and correct any harassing behavior timely, and (2) the complaining employee failed to take advantage of the employer's safeguard measures (108, 109). Similarly, the *Sex Discrimination Ordinance* of Hongkong SAR has also established employer liability provisions. Section 46(1) of the ordinance states that “anything done by a person in the course of his employment shall be treated…as done by his employer as well as by him”. The employer's lack of knowledge or disapproval is no defense. However, under section 46(3), it shall be a defense for employers if they have taken “such steps as were reasonably practicable” to prevent employees' discriminatory behaviors (110).

In mainland China, as already mentioned, national and local legislations have not established specific employer liability rules for workplace sexual harassment. This problem may explain why, among the sample disputes, only one alleged victim brought a vicarious liability claim against her employer. While, in theory, victims may seek relief under the general provisions of tort law, the abstractness of the general clauses can cause confusion: Under what circumstances should the employer bear liabilities? Should the employer assume share responsibility, joint and several responsibilities, or supplementary responsibility with the perpetrator?

In a high-profile lawsuit where a celebrity social worker and the organization he worked for were sued, the court held that the organization should bear no vicarious liability, even though the organization failed to adopt an anti-harassment policy (111). When the costs of violating laws are minimal, employers may lack incentives to comply.

#### The difficulties in proving sexual harassment remain unsolved

The difficulty of proving a sexual harassment case is a common issue in the sexual harassment laws worldwide. First, as can be seen from sample cases collected in this article, since sexual harassment often occurs in hidden places and usually does not cause visible physical damage, it is difficult for victims to provide physical evidence and witness statements. Second, since employers and courts lack understanding of the emotional symptoms of sexual harassment, they tend to discount the credibility of an emotionally unstable victim (112). In addition, there is the issue of evidentiary inequality. Research shows that courts are more likely to exclude evidence submitted by employees in workplace discrimination claims (113).

Current laws and regulations in China have not provided specific rules regarding the difficulties in proving sexual harassment. Recent judicial rulings show that some courts set the bar too high for what constitutes sexual harassment. For example, some judgments require the alleged victim to show evidence of immediate resistance. Similarly, All-China Women's Federation, in its *Guidebook for Prevention and Control of Sexual Harassment in the Workplace*, also states that the victim needs to express “explicit objection at least once” to prove sexual harassment (107). Such an approach is questionable—when the harasser has power over the victim, the victim may not dare to resist. An online survey initiated by *Zhengzhou Evening News* and *Sina News* showed that, only 11.7% of the respondents (*n* = 22,166) chose to “strongly resist” after being sexually harassed (114). It can be seen that in the case of workplace sexual harassment, Chinese courts have not fully understood the victim's lack of resistance does not mean consent. Besides, this article is concerned that some courts in mainland China are giving too much deference to the police. The standard of proof in criminal proceedings is higher than in civil lawsuits. Courts simply deferring to the results of the police investigation in civil disputes can lead to inadequate protection of victims.

#### The lack of legal protection for victims who are not in a traditional employment relationship

The booming digital economy in China has offered new job options. Many choose to become semi-independent workers (115). Since Chinese law has long regarded subordination as the primary element of an employment relationship, influencers and other semi-dependent workers are usually considered independent contractors.

As has been shown in recent lawsuits litigated in Chinese courts, those who are not in a traditional employment relationship, such as social network influencers, can also be vulnerable to work-related sexual harassment. According to article 2 of the *Convention Concerning the Elimination of Violence and Harassment in the World of Work*, workers shall have the right to a harassment-free work environment irrespective of their contractual status. By contrast, since current Chinese anti-harassment laws only require “employers” to prevent and correct workplace sexual harassment, how to sufficiently protect those who are not in a traditional employment relationship from workplace sexual harassment becomes a new challenge.

Another related issue is sexual harassment in remote work. Since the COVID-19 pandemic, full or partial lockdown measures have been applied to reduce transmission of the virus (116), which has accelerated the trend of working remotely (20, 29, 117). Remote workers have a different work environment than traditional employees but can still be subject to work-related sexual harassment (30). As remote work results in blurred work and life boundaries, how to define “workplace”? In remote work, where employers have less control over the workplace, have their obligations to prevent workplace sexual harassment changed? It is necessary to address these questions, although disputes involving remote workplace sexual harassment have not yet been litigated in Chinese courts.

## Law amendment underway: A new chapter or an old story?

China is now proposing to amend its *Women's Rights Protection Law* and has issued the second draft of the revision in April 2022 (118). Although the *Women's Rights Protection Law* only applies to women, its amendment is bound to affect all sexual harassment cases. Will the upcoming new laws effectively fill the gaps in the current legal system?

One of the highlights of the draft law is that it has proposed a new legal definition of sexual harassment. Article 25 of the draft law states that: “Sexual harassment against the will of women is prohibited in the following ways: (1) Verbal remarks with sexual connotations; (2) Inappropriate and unnecessary physical behavior; (3) Displaying or disseminating images, texts, messages, voices, videos, etc. with obvious sexual meaning; (4) Expressing or implying that certain job benefits will be granted in exchange of intimate relationship or sexual relationship, through taking advantage of his position or a superior-subordinate relationship; (5) Other situations that should be considered as sexual harassment.” Compared to the rules in the *Civil Code*, this provision may serve as a guideline by enumerating common types of sexual harassment behaviors. However, the statutory language of the draft law still does not explicitly outlaw hostile environment harassment with no specific target, which falls short of international standards.

Article 27 of the draft law aims to delineate employers' responsibilities. The first paragraph of the article stipulates that: “Employers shall take the following measures to prevent and stop sexual harassment of women: (1) To formulate rules and regulations prohibiting sexual harassment; (2) To appoint personnel responsible for handling complaints; (3) To carry out education and training programs to prevent and stop sexual harassment; (4) To take necessary security measures; (5) To set up complaint hotlines or mailboxes; (6) Establish and improve grievance procedures, handle disputes promptly and protect the parties' privacy; (7) other reasonable preventive and corrective measures.” The second paragraph of the article adds that “the provisions of the preceding paragraph shall apply to new business forms such as the platform economy”. This provision can set more straightforward compliance guidelines and better protect workers in non-traditional employment relationships.

Most worryingly, although the draft law has stipulated employer liability provisions, the liability is too slight. According to the second paragraph of Article 87 of the draft, if an employer fails to prevent sexual harassment and “results in violation of women's rights and interests or bad social impact,” “the higher-level organizations or the competent department shall order it to make corrections”. The deterrent effect of the liability of “making corrections” is far from sufficient.

Taken together, it remains doubtful whether the proposed law amendments will bring breakthroughs. Of course, it would not be fair to blame all the problems on the judges and drafters of law. Legislation and judicial decisions are inevitably subject to a region's cultural, economic, and political atmosphere. In China, although the Constitution has long declared equality between men and women, the problem of gender inequality still has a long way to go. According to data published by China's national statistics bureau, the sex ratio at birth (1.12 males per female in 2017), the sex composition of members of the National People's Congress (with 24.9% female in 2018), and the sex composition of members of trade unions (with 38.5% female in 2018) remain biased (119). *2021 Chinese Women's Workplace Status Survey Report* shows that only 53.1% of male respondents believe that women can be qualified as the company's senior leaders, and 55.8% of female respondents have been asked about their marital status when applying for jobs (120). Moreover, although scholars have long called for Chinese women's organizations to provide legal aid (121), the sample cases did not show that organizations such as the All-China Women's Federation had offered help. Ultimately, only by continuing to advance gender equality can anti-sexual harassment legislation and judicial practice achieve substantial breakthroughs in China.

## Conclusion

Sufficient legislation and vigorous judicial enforcement are essential in eliminating workplace sexual harassment and raising public awareness of gender equality. Although many sexual harassment victims remain silent for complex reasons, the legal system should at least provide a friendly judicial environment and accessible legal remedies for victims who choose to speak up and go to court. Since the passage of its first national law prohibiting sexual harassment in 2005, China has been improving its laws and regulations over the years to address workplace sexual harassment. However, through reviewing current legal safeguards and recent case rulings, this article suggests that existing legislative and judicial responses have drawbacks that shall not be ignored.

## Policy recommendations

Based on the findings of the research, this article proposes the following recommendations for future law revisions and judicial practice:

- To clarify the definition of sexual harassment further, to at least include hostile environment harassment with no specific target, and optimally, to cover gender-based harassment that is not aimed at sexual cooperation;
- To re-frame sexual harassment as both a violation of dignity and a form of gender discrimination;
- To provide guidelines on what reasonable measures employers are required and recommended to take to prevent and correct work-related sexual harassment;
- To provide employers with guidelines for ensuring due process when handling complaints;
- To specify when and how employers will be held vicariously liable for workplace sexual harassment incidents;
- To further alleviate the difficulty of proving sexual harassment in civil proceedings;
- Ensure workers who are not in a traditional employment relationship shall enjoy the right to a harassment-free work environment.

Ultimately, if Chinese policymakers are determined to turn the tide against sexual harassment, as has been emphasized in national policy, there is a need to promote gender equality continuously.

The policy recommendations presented above have implications beyond the context of the Chinese legal system. Not only in mainland China but in other jurisdictions where gender inequality remains high, it is essential to re-conceptualize sexual harassment as both dignitary harm and a form of discrimination, and to establish clear compliance guidelines for employers on measures to prevent and stop sexual harassment at work. In addition, how to ease the difficulties for employees to prove a sexual harassment case and how to adequately protect remote, flexible workers from sexual harassment are challenging for China and other countries worldwide.

## Data availability statement

The original contributions presented in the study are included in the article/supplementary material, further inquiries can be directed to the corresponding author.

## Author contributions

Conceptualization, methodology, analysis, and writing: HW.

## Funding

This research was funded by Funding for Scientific Research of Hangzhou Normal University (Granted Number: 4025C50218204077).

## Conflict of interest

The author declares that the research was conducted in the absence of any commercial or financial relationships that could be construed as a potential conflict of interest.

## Publisher's note

All claims expressed in this article are solely those of the authors and do not necessarily represent those of their affiliated organizations, or those of the publisher, the editors and the reviewers. Any product that may be evaluated in this article, or claim that may be made by its manufacturer, is not guaranteed or endorsed by the publisher.
